# A Score-Based Game Approach Considering Resource Heterogeneity and Social Dynamics for Traffic Optimization in Social IoT Networks

**DOI:** 10.3390/s25072297

**Published:** 2025-04-04

**Authors:** Muhammad Muneer Umar, Ali F. Almutairi, Shafiullah Khan

**Affiliations:** 1 Institute of Computing, Kohat University of Science & Technology, Kohat 26000, Pakistan; 2 Electrical Engineering Department, Kuwait University, Safat 13060, Kuwait; 3 College of Engineering and Energy, Abdullah Al Salem University, Khaldiya 72303, Kuwait; 4 College of Computing and Systems, Abdullah Al Salem University, Khaldiya 72303, Kuwait

**Keywords:** social internet of things, routing in SIoT, selfish node management, trustworthy routing

## Abstract

The incorporation of human-like social concepts into the Internet of Things (IoT) has given rise to the paradigm of Social IoT (SIoT). In these networks, objects autonomously form social relationships to enhance network scalability in information and service discovery, focusing on their own benefits. However, social likeness or dislikeness among nodes can result in selfish behavior, adversely affecting network performance. Existing node stimulation mechanisms primarily focus on ad hoc and IoT networks, emphasizing topological structures and traffic patterns, while overlooking the social and behavioral factors crucial to the SIoT. This work proposes a novel node stimulation scheme for the SIoT that incorporates both social and behavioral characteristics and network topology. The mechanism employs a virtual currency-based game to incentivize cooperation by considering parameters such as proximity, energy levels, buffer size, correlated relays, and data quality. Additionally, social factors—including social preference, node importance, interaction history, and the probability of vital data transfer—are integrated into the decision-making process. Simulation results demonstrate that the proposed mechanism outperforms existing approaches in terms of energy efficiency, throughput, packet delivery ratio, and end-to-end delay, making it a robust solution for improving cooperation and performance in SIoT networks.

## 1. Introduction

A part of the Internet of the future, the Internet of Things (IoT) is a dynamic global network infrastructure that may self-configure based on standards and compatible communication protocols, giving both virtual and physical objects unique identities. These networks connect a wide range of everyday objects, often referred to as “things,” to the Internet. This connectivity allows them to collect, process, and exchange data using various types of protocols. By implementing IoT solutions, various industries are automating tasks and freeing human workers for more complex activities [1,2]. These networks consist of a variety of devices, making them highly heterogeneous in nature. This heterogeneity is a major challenge for effective network functioning. For example, an actuator node with a lot of power may require more data processing than a resource-constrained sensor node with a limited battery life [3,4].

The Social IoT (SIoT) networks extend beyond the basic functionalities of IoT by allowing devices to establish social interactions with each other. These interactions can be based on physical proximity, data transfer history, and likeness or dislikeness. These social behaviors offer various benefits for a cooperative environment and smooth operations of nodes [5]. The nodes of these networks are designed to function intelligently and make decisions based on their own interests. However, the self-autonomy of nodes introduces new challenges. Self-decision-making behavior can lead to situations in which nodes act selfishly. Selfish nodes do not cooperate with others during network operations to preserve their own resources [5].

Selfish nodes can be categorized into various forms. Some examples are individual nodes saving resources by refusing to forward data, social groups within the networks where the cooperation is selective, or adapting a non-cooperative behavior for all the nodes in the entire network. Such behaviors can significantly affect poor performance, causing significant delays, packet loss, and degradation of the overall efficiency of the network. However, these nodes may take advantage of relaying the services of others during their data transfers [6]. Various research proposals have been made to stimulate selfish nodes in such types of networks [7].

The heterogeneous resource levels of nodes in the SIoT need to be properly addressed. Resources like buffer size, and energy availability can considerably affect a node’s ability to participate in data communication. While various research proposals have been made to stimulate cooperation among selfish nodes, most of them focus on topological and traffic-based mechanisms. However, they do not fully integrate both the social aspects of nodes (such as trust, relationships, and behavioral patterns) and the heterogeneous nature of SIoT networks in terms of energy, buffer size, and communication capabilities. This lack of integration leads to inefficient cooperation incentives and suboptimal network performance. To address this gap, there is a need for a routing mechanism that not only stimulates cooperation among nodes through a virtual currency system but also considers both the social and resource heterogeneity aspects of the SIoT. By integrating social relationships and resource-based constraints into a scoring-based game, the mechanism can ensure fairness in relaying services while improving the overall network performance.

In this work, we propose a novel routing mechanism for SIoT networks that uses a virtual currency, referred to as a score. Each node in the network maintains a score by selling its relaying service to other source nodes. Our proposed mechanism stimulates cooperative behavior while ensuring optimal performance within the network by integrating social relationships and resource heterogeneity into a score-based game. Heterogeneous nodes are represented by differences in energy levels, computational capabilities, and social preferences, which influence their role in routing. Social interactions are incorporated via a scoring-based mechanism, where nodes build trust and cooperation based on past interactions. A node’s importance is determined by its energy, available neighbors, and historical behavior, and its social preference impacts relay selection. The routing process dynamically adapts by considering both network and social factors, ensuring incentivized cooperation and efficiency in the SIoT environment. This approach encourages nodes to participate in data communication based on their capabilities and fosters a collaborative environment in which nodes build trust and rely on each other for efficient data forwarding.

The remainder of this paper is organized as follows: Section 2 provides a review of related work in the field of data traffic management for SIoT networks in the presence of selfish nodes. Section 3 establishes the preliminary concepts and definitions needed for the proposed mechanism. Section 4 describes the details of the proposed mechanism. Section 5 presents the details of the simulation scenarios. To evaluate the effectiveness of our approach, Section 6 presents the simulation results that compare our approach with existing solutions. Finally, Section 7 concludes the paper by summarizing the key findings and outlining the potential opportunities for future research.

## 2. Related Work

One of the key characteristics of the IoT is the presence of heterogeneity. There are a vast variety of nodes within the network. These nodes can have very different resource sets like processing power, battery life, and storage, and perform diverse operations. The variety of devices, communication methods, and data formats in the IoT create obstacles to large-scale integration. This “heterogeneity” makes it challenging for devices to work together. The authors of [3], explore these challenges, like devices with different capabilities or using unique data formats. Overcoming these hurdles is vital for achieving the full potential of an interconnected IoT world. Moreover, the diverse traffic patterns generated by such heterogeneous nodes are a rising challenge [8].

A social-based watchdog system [9] to detect selfish nodes in opportunistic mobile networks builds on the conventional watchdog mechanism by integrating social awareness into the detection process. Unlike traditional watchdog systems that rely solely on direct observation of node behavior, this approach incorporates social relationships and interactions to improve the accuracy and adaptability of selfish node detection in dynamic network environments. primarily detects selfish behavior by monitoring and recording packet forwarding actions in opportunistic mobile networks (OMNs). It relies on social ties and reputation scores to isolate non-cooperative nodes. In contrast, our proposed SIoT mechanism leverages dynamic social preferences, honesty-based evaluations, and reward-based mechanisms to differentiate between selfish and malicious nodes. Instead of relying solely on historical forwarding behavior, our approach adapts node selection strategies based on data quality, social interactions, and network conditions, ensuring more efficient routing and improved cooperation in heterogeneous SIoT environments.

SIoT is a variant of the IoT in which nodes act socially, similar to humans. The nodes are considered smart enough to autonomously decide their operations. Such nodes build up their social networks to achieve their goals, such as resource preservation, performance optimization, and obtaining quality services and data. There is a lack of clear procedures and mechanisms for handling the social and behavioral aspects of SIoT network nodes [10].

Selfishness in ad hoc networks has been addressed in many research articles. The authors of [11] reviewed the existence and effects of selfishness in ad hoc networks. The techniques used for a cooperative environment in such networks and future challenges are also discussed in this work. These techniques are classified into detection, trust, game-based, and incentive-based mechanisms.

Virtual currency can be used to incentivize cooperation in the IoT by rewarding nodes that contribute resources and penalizing those that act selfishly. This approach of virtual currency has been utilized by many researchers. Xuemin et al. [12], propose a solution to address challenges in networks where nodes exhibit social selfishness due to limited resources and social connections. It introduces the Resource-Constrained and Socially Selfish-Based Incentive Algorithm (RSIA) that utilizes virtual currency to encourage cooperation by rewarding message forwarding and penalizing uncooperative nodes. Moreover, various game theory approaches based on virtual currencies have been proposed to handle non-cooperative environments in the SIoT and similar networks. The work, Game-theoretic Incentive Scheme for Social-aware rOuting (GISSO) [13] proposes a scheme that aims to encourage cooperation in message forwarding within mobile social networks where nodes might act selfishly due to limited resources. GISSO uses game theory to create incentives for nodes to participate in routing by rewarding those that forward messages and potentially penalizing those that do not forward messages.

A game theory reward-based mechanism [14] has been proposed for the stimulation and management of selfish nodes in wireless sensor networks. A bargaining game is used to handle virtual currencies referred to as scores. The nodes are stimulated to participate in the network using these scores and are penalized using a card system. This work has considered various parameters when forming the game. Another game theory approach proposed by Nobahary et al. [15] utilizes a hierarchical structure in which nodes play repeated games to determine their reputation and identify those that do not forward data packets as expected. The primary objective of this work is to identify nodes that do not cooperate within the network. A study [16] combined fuzzy systems and game theory to detect and mitigate selfish nodes in the IoT. Fuzzy logic evaluates node behavior based on varying degrees of selfishness, thereby allowing flexible classification. The game theory model incentivizes cooperation by balancing rewards and penalties to ensure fair participation. The system dynamically updates reputation score-based on historical behavior and adapts to evolving selfish strategies. By integrating fuzzy decision-making and strategic interactions, this approach enhances network stability and fairness. While these game-based approaches effectively manage selfish behaviors and improve network efficiency, they primarily focus on optimizing network performance. They often overlook the deeper aspects of social connectivity, such as dynamic trust evolution and subjective social influences.

Reputation-based systems have also been introduced in various mechanisms to address the issue of selfish nodes. Xiao et al. [17] proposed DSNDA, which detects selfish nodes using forwarding intention, reputation, and confirmation messages. However, it overlooks social selfishness and may misclassify cooperative nodes due to caching issues. Xiong et al. [18] introduced CCSDA, combining reputation assessment and interaction-based detection. It reduces energy consumption but does not fully address social selfishness, leading to potential misjudgments.

Community-based mechanisms are also presented to handle the selfishness of nodes in similar networks. A community-based mechanism [7] proposes a technique that uses the honesty levels of nodes by accessing their interaction frequencies to create “credible communities” in the SIoT. Nodes are evaluated based on their honesty, and trustworthy nodes are selected as leaders to incentivize good behavior and potentially punish dishonest nodes. This approach aims to increase cooperation and improve overall network performance. Another community-based work, SOS (Socially Omitting Selfishness) [19], focuses on selfishness in smart and connected communities within the IoT. In this work, nodes elect leaders for data forwarding based on their energy levels and past cooperation. A fair economic model (VCG) incentivizes contributors and discourages selfish behavior, while monitoring ensures that everyone plays by the rules. Some community-based systems have also been proposed to assess inter- and intra-community trustworthiness in the IoT. Su et al. [20] introduce a role-based dynamic trust model to evaluate node trustworthiness while resisting malicious attacks. The model assesses nodes based on their roles and interactions within the community to ensure adaptive trust updates. Additionally, it employs an attack-resilient mechanism to mitigate the manipulation of trust. This approach enhances security by maintaining a robust trust evaluation system against collusion and misbehavior in IoT environments. However, this work primarily focuses on detecting malicious behavior and does not explicitly address selfishness influenced by social ties among nodes.

In another article [21], the authors propose an energy-aware resource allocation technique for Mobile Internet of Things (MIoT) networks, combining selfish node ranking and ant colony optimization (ACO) to reduce energy consumption and end-to-end delays. The proposed method outperforms existing algorithms like WOA and TPRA, addressing challenges such as node heterogeneity and selfish behavior. This study emphasizes the need for adaptive strategies to enhance MIoT performance and offers insights into future research. However, it does not address social preferences or likeness among network nodes.

Trust management plays a vital role in such networks. Bao and chen [22] explore the challenges of trust management in a heterogeneous environment, suggesting reputation and social connections as potential tools to ensure trustworthy interactions between devices. A trust-based quantitative model is proposed in article [23] for node behavior detection in the IoT. The work focuses on evaluating direct interactions, recommendations from other nodes, and historical data to assess a node’s trustworthiness. This comprehensive approach helps to identify unreliable nodes while minimizing energy consumption. Another machine learning trust management system was proposed by Jayasinghe et al. [24]. This method extracts relevant trust features from the environment and utilizes unsupervised learning to categorize interactions as trustworthy or not. The core of the approach involves a Support Vector Machine (SVM) model that learns to predict the overall trust value of interactions based on the extracted features. In another article [25], the author addresses selfish node detection in MANETs by considering both fixed and unpredictable mobility. He proposes a stochastic Markov model to reduce storage overhead and employs a consensus and discounting operator to mitigate biased viewpoints. Digital signatures based on public keys are used to ensure non-repudiation. The author assesses the system’s efficiency by analyzing its impact on the trust computation, throughput, complexity, and resource consumption. Authentication is not involved, assuming that all participating nodes are pre-trusted.

Zhang et al. [26] propose a resource-based dynamic pricing strategy combined with blockchain to incentivize data forwarding in socially aware networks. Their Forced Forwarding Incentive Algorithm (DFIA) integrates pricing mechanisms, trust management, and social interactions to mitigate selfish behavior and enhance cooperation among nodes. Blockchain ensures secure and tamper-resistant transactions, while social trust mechanisms improve data transmission efficiency. However, this approach introduces computational complexity and potential scalability challenges.

In a paper [27], the authors introduce an auction-based routing mechanism designed to identify and manage selfish nodes within Delay-Tolerant Networks (DTNs). By employing an optimal auction mechanism, the scheme encourages cooperation among nodes, thereby enhancing the overall efficiency and reliability of data transmission in DTNs. The auction mechanism assigns a bidding value to each node based on its past forwarding behavior and its willingness to cooperate. Nodes with higher bids are prioritized for data transmission, ensuring that reliable and cooperative nodes are rewarded while selfish nodes are penalized by reducing their participation in the network. The scheme relies on accurate bid evaluation, which may be vulnerable to strategic manipulation by selfish nodes that falsely appear to be cooperative.

In addition to the proposed work, network topology and other factors influence performance. For example, rumor spread studies [28,29] show how network structure impacts information flow and control, similar to the effect of topology on data transmission in the SIoT. Similarly, environmental heterogeneity [30] also plays a crucial role in system dynamics. For instance, studies on memory-based diffusion in predator-prey models demonstrate how heterogeneous environments facilitate species survival, just as network structures impact data flow and interaction patterns in the SIoT.

While existing research offers valuable insights, there is a need for more comprehensive solutions that effectively address the following limitations:

- Resource Heterogeneity

Many existing approaches focus on energy as a single resource and do not adequately account for the diverse resource profiles of nodes in SIoT networks. Some nodes may have limited energy but do not require frequent operation, resulting in a low energy consumption ratio. Moreover, communication bandwidth, storage buffer, etc., should also be considered [31].

B. Static Reward Structures

Some game-theoretic approaches utilize fixed reward structures that may not adapt to dynamic network conditions or to varying resource levels. Moreover, nodes may have varying initial resource endowments [32].

C. Complexity of Trust Mechanisms

Establishing and maintaining trust mechanisms in the SIoT can be computationally expensive and may not be suitable for all applications. Lightweight trust mechanisms that balance security and computational efficiency are crucial for large-scale SIoT deployments. These mechanisms enable nodes to assess the trustworthiness of others without incurring significant overhead [33,34].

D. Minimal Consideration of Social Behavior

Most existing mechanisms focus solely on the typical structural aspects of similar networks. Social affiliations and likenesses of the nodes are not considered. Ignoring social aspects can lead to inefficient resource utilization across the entire network. Mechanisms that consider social connections can incentivize cooperation and promote better load balancing among nodes [7].

E. Limited Consideration of Data Quality

For an efficient data traffic and selfishness management scheme, the quality of data delivery must be considered [35]. The efficiency of routes, quality of data transfer, probability of vital data, and history of successful interactions among nodes significantly influence the performance of networks.

While existing research has made significant progress in addressing selfishness in the IoT, SIoT, and relevant networks, several critical limitations persist. Most prior studies primarily focus on energy constraints while overlooking other resource heterogeneity factors, such as bandwidth, processing power, and storage. For instance, many game-theoretic incentive mechanisms [11,12,13] utilize fixed reward structures that do not adapt to dynamic network conditions, leading to inefficiencies in terms of long-term cooperation. Similarly, reputation-based trust mechanisms [16,17,18] often introduce high computational overheads, making them impractical for large-scale SIoT environments. Another major gap in the existing literature is the limited consideration of social behavior in SIoT networks. While some mechanisms, such as community-based frameworks [7,15], leverage social interactions, they primarily focus on node honesty rather than social preferences and likeness among nodes. This neglect of social affiliations can result in suboptimal cooperation incentives and inefficient resource utilization.

The proposed approach addresses these limitations by introducing a dynamic incentive mechanism that adapts rewards and penalties based on the network conditions, thereby ensuring sustained cooperation in resource-constrained environments. The framework considers multiple resource constraints beyond energy, integrating bandwidth, processing power, and storage availability into the incentive structure. Additionally, we incorporate social awareness into the selfish node detection process by evaluating nodes’ past interactions and preferences, ensuring that cooperative behavior is encouraged through socially driven incentives. Moreover, the proposed framework integrates historical data quality and successful interaction rates to optimize the data routing and delivery. This ensures that not only are selfish nodes identified, but also data transfer efficiency is also maximized.

## 3. Preliminaries

In traditional and typical ad hoc networks, nodes operate on predefined procedures. However, SIoT networks involve intelligent nodes that can make their own decisions. This self-deciding behavior can lead to the act of selfishness. Selfish nodes prioritize their own resource conservation over the network goals. For instance, a node with a limited battery life might refuse to forward data packets to other nodes to preserve its own energy. This requires a mechanism that stimulates the cooperative behavior of all nodes within the network.

This section discusses the fundamentals needed to design the proposed mechanism in SIoT networks. This work is influenced by our previously proposed reward-based mechanism [14].

- Network Model

We consider an SIoT network consisting of a set of smart nodes, denoted by N={1, 2, ..., n}, where n represents the total number of nodes in the network N. These nodes operate within a predefined geographical area and communicate with each other using the common underlying communication protocol IEEE 802.15.4 [36]. The specific protocol choice may depend on factors like network size, data transfer requirements, and power consumption constraints. However, for our game framework, we focus on the logical, social, behavioral, and topological aspects of the network and assume a reliable underlying protocol that enables data exchange between nodes. It is also assumed that the nodes do not exchange false routing information with each other.

B. Classification of Nodes

SIoT and similar networks are comprised of heterogeneous nodes with varying capabilities. The nodes can be classified as highly constrained, balanced, or high-capacity. Moreover, the nodes can autonomously adjust their social likeness or cooperation levels.

C. Nodes’ Resources

The resource capacity of a node i can be determined by its energy ratio and residual resource. We can consider the energy consumption ratio, ${ECR}_{i},$ of node i by using Equation (1):

(1) $${ECR}_{i}=\frac{E_{i}^{t}}{\Delta E_{i}^{T}}$$

where $E_{i}^{t}$ is the percentage of the current energy of a node i at time t. $\Delta E_{i}^{T}$ is the percentage variation in the energy of node i in time period *T*. The residual buffer size is given by Equation (2) below:

(2) $${BF}_{i}={BF}_{base}+k\;(L_{i}-L_{avg})$$

In the equation, ${BF}_{base}$ is the base buffer size assigned to nodes, k is a scaling factor that determines the extent to which the buffer size changes in response to changes in traffic load. $L_{i}$ and $L_{avg}$ are the traffic loads on nodes i and the average traffic loads in the network, respectively.

D. Selfish Nodes

A node can be considered selfish if it does not provide relaying services to other nodes’ data traffic. It is possible to determine the node i’s participation by adding up all the relaying requests made to it and its neighbors. Equation (3) can be used as follows:

(3) $${Part}_{i}=\frac{{nPkFwd}_{i}}{{nRlyReq}_{i}}\left[\sum_{j=1}^{n\left(neigh\right)i}{nPkFwd}_{j}-\sum_{j=1}^{n\left(neigh\right)i}{nNPkFwd}_{j}\right]\;$$

${nRlyReq}_{i}$ stands for total relay requests, while ${nPkFwd}_{i}$ indicates the total number of packets forwarded in the above equation. The method also adds all the neighbors’ nPkFwd. In addition, some neighbors may forward packets that are not requested by node i. This number is therefore subtracted from the total number of packets forwarded.

E. Physical Proximity

Unlike traditional IoT and WSNs, SIoT networks allow nodes to establish social connections with each other. These connections can be based on various factors, including the physical proximity of the two nodes.

The physical proximity between nodes i and j can be represented by the distance between them. The distance between two nodes is computed using the received signal strength indicator (RSSI) mechanism. This value is computed using Equation (4) and is used to determine the physical proximity of the nodes [37].

(4) $$D_{i,j}=P_{i,j}^{-1}\left(d\right),\;\;P_{i,j}\left(d\right)=\frac{p_{i}G_{i}G_{j}\Lambda^{2}\;}{{\left(4\pi \right)}^{2}d^{2}}\;\;$$

where the transmitter and receiver nodes are represented by i and j, respectively. The transmission power is represented by $p_{i}$, whereas the antenna gains of the two nodes are represented by $G_{j}$ and $G_{j}$, respectively. The wavelength of the transmission signal, expressed in meters, is indicated by Λ. When the inter-node distance is *d*, the receiving power at node j is $P_{i,j}$.

F. Co-Located Relay Nodes

During data forwarding, some nodes receive relaying requests from almost similar sources all the time due to their placement. This type of situation is very common in densely deployed nodes, which are referred to as co-located relay nodes (CRN) in this work. Any two nodes i and j can be considered as members of a set *k* of CRNs, if the following probability-based condition in Equation (5) is met:

(5) $${CRN}_{k}=1-{\left[1-\lambda \times \frac{P_{dest}}{A}\right]}^{S}\;\;$$

${CRN}_{k}$ represents a set of nodes that have the probability of receiving similar route Requests (RREQs). λ is the attraction factor 0≤λ≤1. This factor captures the tendency of sources to prefer specific co-located groups over others. A value of 1 indicates that sources are equally likely to choose any co-located group, while a value closer to 0 suggests a stronger preference for certain groups (e.g., based on historical success or real-time congestion). $P_{dest}$ is the probability of having the same destination nodes in the RREQ. The area covered by the CRNs is denoted by *A*, and *S* represents the total number of data sources i in the network. This parameter can be considered under the segment of social aspects.

CRNs are identified as spatially close nodes that share similar connections and frequently receive relay requests from the same sources. To optimize relay selection and prevent redundant transmissions, the proposed scheme prioritizes nodes based on their availability, historical success rate, and congestion level. Among the CRNs, the node with the lowest forwarding load and highest successful transmission rate is selected, ensuring efficient and balanced data forwarding. This approach minimizes packet collisions and enhances the overall network performance.

G. Importance in Network

Another social aspect of a node is its individual importance in the network. Each node in the network possesses some level of importance, based on its locality. Nodes with a smaller number of neighbors can be considered more important because such nodes are the only option for relaying traffic. Dense nodes can provide multiple alternate routes for data traffic. The importance of a node can be computed using Equation (6) as follows:

(6) $${imp}_{i}=\frac{E_{i}}{{n(CRN}_{k})\sum_{j=1}^{{n(CRN}_{k})}E_{{CRN}_{k}}}\;$$

A higher value of imp of a node i indicates a lower probability of a lower node energy, a fewer number of closed neighbors, and/or lower energies of these neighboring nodes. All we have to say is that the energy level of a node directly correlates with its relevance, while the number of closely located neighbors and their energy levels have an inverse relationship. If a node is located near some other nodes that could also take the same relaying requests, then the node will have low importance.

H. Data Transfer History

The data transfer history between nodes i and j can be captured by $H_{i,j}$, which represents the number of successful data exchange events between them in the past. A higher $H_{i,j}$ signifies a more positive data sharing history, leading to a stronger social connection. The data exchange history for a period T can be computed by Equation (7):

(7) $$H_{i,j}=\frac{{DDV}_{i,j\;}}{T}\times \;\;\;\frac{100}{{DDV}_{max}}$$

In this equation, ${DDV}_{i,j}$ represents the successfully delivered data volume in terms of packets between nodes i and j over a time period T.

Nodes having higher $H_{i,j}$ can be considered closely bounded nodes, leading to stronger social connections; such nodes may require frequent interactions between them. During relaying services, such nodes should be given more privileged attention while deducting the score for relaying services.

I. Vital Data for a Node

Nodes in a network often act as recipients of information from various other nodes. While some data might be considered routine or less important, other transmissions can be crucial for the node’s functionality. These critical data streams hold important information that directly affects a node’s ability to perform its tasks or achieve its goals. Such a connection should always be maintained with the source node. To represent the overall probability of destination node j receiving vital data from the source node i, Equation (8) can be used:

(8) $$P_{j,i}^{vital}=P_{i}^{data}\times P_{i}^{vaitality\;|\;data}$$

$P_{i}^{data}$ represents the event in which node i transmits any data to node j after the route has been established, and $P_{i}^{vaitality\;|\;data}$ represents the conditional probability that the data transmitted from node i are actually vital for node j during data transmission. This mainly relies on the type and nature of the data that node i is typically transmitting and how well it is aligned with the needs of node j.

J. Prediction of Enough Score

In the proposed work, nodes operate autonomously, making decisions without constant intervention. A crucial aspect of this autonomy is the ability to effectively manage earned scores. These nodes constantly evaluate their score balance to determine whether it is sufficient to cover the costs of future data transmission. To assess the score sufficiency for future transmissions, the node i leverages a probability calculation based on its transmission history. This is represented by Equation (9):

(9) $${P(t)}_{i}^{enoughscore}=1-\frac{C_{i}^{t}}{{⋃Score}_{i}-\sum {Hd}_{i}}$$

In this equation, we take the average transmission cost, $C_{i}^{t}$, of node i for the time period t. This value is divided by the difference between the union of all scores, ${⋃Score}_{i}$, earned and the summation of all historical deductions $\sum {Hd}_{i}$ of node i for time period t.

K. Quality of Data Transmission

Furthermore, the quality of the dataflow is also very important. Two nodes may have weak connections in terms of lower bandwidth, higher error rate, or higher latency. The quality of data transmission between two nodes i and j can be shown by Equation (10)

(10) $$Q_{i,j}=\frac{\omega_{pdr}PDR}{\omega_{ber}BER\;\;\;\;\;\;\;\;\;}+\frac{\omega_{b}B}{\omega_{lat}LAT}$$

In this equation, we have normalized values (0 to 1) of the packet delivery ratio (PDR), bit error rate (BER), bandwidth (B), and latency (LAT). The weights for each item are represented by their respective *ω*s.

## 4. Proposed Mechanism

The proposed mechanism leverages social dynamics to optimize routing decisions in wireless decentralized ad hoc networks by incorporating node importance as a key factor. Unlike traditional approaches that rely solely on topological structures and traffic patterns, our method integrates social relationships, node energy levels, and neighborhood density to enhance the routing efficiency. The energy level of a node is directly correlated with its importance in the network, as higher energy nodes are more capable of relaying data. Moreover, a node with many closely located neighbors that can also handle relaying requests has lower individual importance, reducing redundancy and balancing energy consumption across the network. This socially aware approach allows for dynamic adaptation in environments using Bluetooth, Wi-Fi, or ZigBee, where factors such as radio signal energy adaptation, connectionless and connection-oriented modes, and frequency hopping can influence the network performance. By considering these social and resource-driven factors, the proposed mechanism improves routing decisions, leading to better network life and enhanced cooperation among nodes.

The major objective of this work is to design a virtual currency-based game for the SIoT that utilizes the social elements inside the network so that selfish nodes can be properly managed. Additionally, nodes with considerably less participation history are adaptively handled by considering the nodes’ proximity and importance inside the network. Moreover, persistent selfish nodes are kept isolated but are always given chances to participate in the network.

This work uses a score-based game to manage traffic flows between nodes during data relaying. This model focuses specifically on the stimulation of nodes toward their participation in network data traffic while considering their resource consumption. During data transmission from a source node over some relay nodes, the source and potential forwarders deal with scores associated with data forwarding. In this way, a scenario of buying and selling is established among the nodes. If a node needs to send its data, it should maintain a sufficient score by helping other nodes with its relaying services.

**Figure 1:**
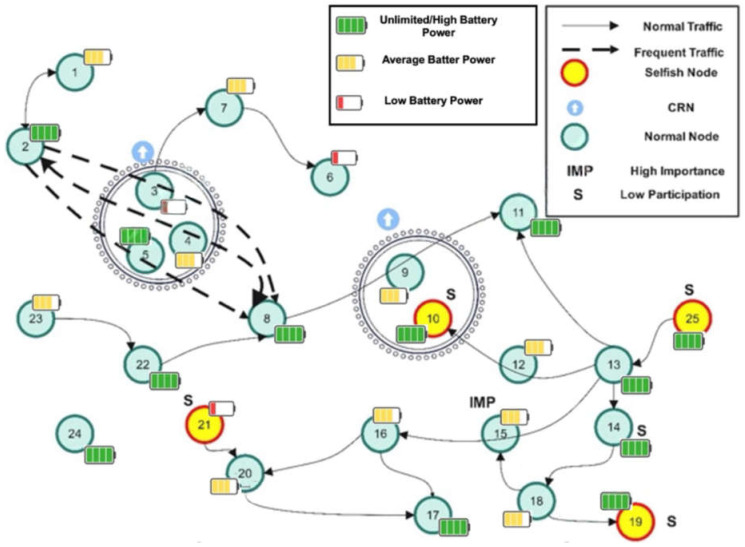
Network model having heterogeneous nodes and social interactions.

Figure 1 shows a network with various types of nodes. These nodes operate on different resource sets and perform diverse functions. The figure illustrates the traffic flow and social connections between nodes, with some nodes interacting more frequently than others. For example, Node 8 has a strong connection to Node 2. These two nodes utilize relaying services provided by Nodes 3, 4, and 5, which are closely located and identified as members of a CRN set.

Node 3 has exhausted its energy and may stop cooperating with Nodes 2 and 8. There might be two reasons for its lower energy condition: (a) it has participated more than Nodes 4 and 5, or (b) it has a lower energy resource from the beginning. Node 10 could be a relay for Nodes 8 and 11, but it does not cooperate, which causes Node 9 to lose more energy. Node 8 solely relies on Node 9 to connect to Node 11. Therefore, Node 10 might have social dislikeness or selfishness. Node 19 also exhibits a low participation level due to its border placement and lack of relaying requests. Unlike Node 10, Node 19 cannot be considered selfish because it does not receive any relay service. Some nodes are identified as critical nodes due to their strategic positioning. Consider the example of Node 15. These nodes either have no CRNs or are surrounded by CRNs with depleted energy reserves. Node 21 is marked as non-cooperative. This might be because this node did not have enough energy and thus adapted selfishness.

In such SIoTs, some nodes may have frequent interactions with others but lack cooperation. These selfish nodes benefit from other nodes’ relaying services without reciprocating. Border nodes may have low participation levels due to their location, resulting in fewer relay requests. However, these nodes cannot be classified as selfish solely because they receive a low number of requests.

This section is further divided into game formation, scoring mechanisms, score-based games and selfish node management.

- Game Formation

This work utilizes a game to incentivize cooperative data forwarding behavior among nodes in an SIoT network. A typical game framework comprises players, strategies, and a payoff function. The nodes in the network can be referred to as players. These players can have various roles. For example, a single node can serve as the source, destination, and data forwarder. These roles can be used to highlight the specific interactions among players when they initiate, receive, or relay data. We can denote the set of players as $N=\{{pl}_{1},\;{pl}_{2},{pl}_{3}\;\ldots ,{pl}_{n}\}$, where n presents the total number of nodes in the network. While maintaining the roles of source, destination, and/or relay, these nodes can operate using a set of strategies. Since the nodes are smart and operate autonomously, they can choose to cooperate, not cooperate, adaptively/conditionally cooperate, and gain more scores. The strategies for a node i can be a set denoted by $S=\{s_{1},\;s_{2},s_{3}\;\ldots ,s_{n}\}$. The set of all possible strategies for all players is denoted as $\Gamma =\{s_{i}^{*}\}$, which represents the complete strategy space of the game. The payoff or utility function plays a vital role in this model for node stimulation and cooperative data forwarding. It determines the utility of each node based on its actions and the actions of other nodes. The nodes use this function to adjust their cooperation level with their resource management. Nodes with active participation levels will maintain a higher level of their score but may consume their energy.

By establishing a framework for player interaction, strategy selection, and negotiation, this game model aims to promote cooperation and efficient data forwarding within the SIoT network. Therefore, the game can be formulated as follows: G={P,S,U}.

The proposed game in this work is a score-based incentive game with elements of cooperative game theory and mechanism design, specifically designed for SIoT networks. It encourages nodes to actively participate in data forwarding by introducing a virtual currency system in which nodes accumulate and spend scores based on their cooperation levels. The game exhibits the characteristics of non-cooperative strategic interactions because each node autonomously decides its strategy of whether to cooperate, conditionally cooperate, or remain selfish. However, it also incorporates elements of cooperative games, as nodes with mutual social preferences can set lower score demands (DS) for each other, thereby demonstrating stronger social ties. The payoff function is defined in terms of accumulated scores, which incentivizes participation while considering resource constraints, such as energy levels and buffer sizes. The game also integrates auction-like mechanics, where nodes “trade” relaying services in exchange for scores, thereby creating a dynamic buying and selling environment. Furthermore, adaptive selfish node management ensures that non-cooperative nodes face isolation while still being given the opportunity to reintegrate into the network. The scoring mechanism dynamically adjusts based on network conditions, node importance, and historical cooperation, making it an adaptive, incentive-driven game model designed for SIoT environments.

B. Scorning Mechanism

A node initiating a data transfer must maintain an adequate score to pay the intermediate nodes for their relaying services. In contrast, the nodes try to earn higher scores by cooperating with others so that they can easily transfer their data.

The demanding score, DS, of each relay node i, is the amount of score that is deducted from the score of the source node. The DS of each node may vary depending on its resource level and social aspects. Two nodes may have very strong levels of interactions and, therefore, may set their DS as low as possible for each other. Conversely, a node having a low buffer size or energy may need high DS to preserve its sacred resources. Table 1 shows the factors influencing the amount of DS for each node

The following Equation (11) can be used for setting the DS of node i for a source node *j*.

(11) $${DS}_{i\to j}=\frac{H_{i,j}\times {ECR}_{i}}{{BF}_{i}\times Q_{i,j}}\times \frac{{SPref}_{i\to j}\times 100}{{n(CRN}_{k})\times AVG\left(\;E_{{CRN}_{k}}\right)}\;$$

where node i is the member of ${CRN}_{k}$. The absolute value of social preference, ${SPref}_{i\to j},$ represents the social likeness or dislikness of node i toward node j. If node i likes node j, then this value is less than 1, otherwise higher for dislikeness or selfishness. This value can be autonomously adjusted by the node. Being a part of the CRN set, node i is also counted, and its energy is considered when taking the average of the CRN node energies.

C. Score-Based Game

Whenever a node initiates data transfer, it generates an RREQ frame that is broadcast to all connected nodes. A typical RREQ contains the source and destination addresses, hop count, request ID, and other control information. The source node piggybacks its score values onto the RREQ, as listed in Table 2.

Upon receiving the RREQ, nodes typically verify the destination address. If the destination address does not belong to the node, it forwards the frame to its neighbors according to a predefined routing algorithm. This algorithm may consider factors such as the distance to the destination, hop count limit, or neighbor information. Each node i, upon forwarding the request for node j, deducts its ${DS}_{i\to j}$ from the source score field. In this process, the source score value decreases with each intermediate node along the route.

Conversely, if the receiving node is the destination node, it first checks the source score. A positive score value indicates that the source possesses an adequate score to compensate for the relay nodes. Therefore, in such instances, the destination node generates the RREP (Route Reply) frame, which is then sent back to the source node. In this RREP, the destination node also includes the value of the source score received in the RREQ, as shown in Table 3. Since it is evident that the destination node may have some social preference for the source node, it also incorporates its SPref so that the source node may adjust its SPref accordingly.

The destination node may receive an RREQ containing a negative value for the source score, indicating that the source does not possess an adequate score to compensate for all the relay nodes. Such a scenario could be deemed suspicious. In general scenarios, selfish nodes cannot maintain their scores by cooperating with other nodes; therefore, such nodes cannot pay the relay node for their data transfers. However, there are other reasons why such nodes may lack an adequate number of scores.

It is evident that certain nodes may need to transmit data frequently while receiving relay requests infrequently. Additionally, a single transmission of data may require one node to handle multiple relay requests. To address this issue, upon a successful connection, a node receives a multiple of DS. The value of DS is multiplied by the total number of relay nodes involved in the route. Therefore, the score of node is updated as shown in Equation (12).

(12) $${Score}_{i}={Score}_{i}+{DS}_{i\to j}\times \alpha$$

The node i updates its score by adding the the calculated DS for node j multiplied by a coefficient α. The coefficient α can be used to balance between incentivizing relaying and avoiding excessive score inflation due to the high number of relays. The total number of relay nodes, $n\left(RlyNodes\right),$ excluding the source and destination nodes, can also be considered.

The proposed routing mechanism operates at the network layer of the OSI model. It utilizes connectionless communication, primarily leveraging UDP for routing control messages. The lower-level protocols used include IP for addressing and packet forwarding, while the underlying MAC and physical layer protocols depend on the specific SIoT deployment scenario.

D. Selfish Node Management

In typical scenarios, selfish nodes are unable to sustain their scores by not cooperating with other nodes. Consequently, such nodes are incapable of compensating for relay nodes for their data transfers.

However, the destination node also takes into account the significance of the source node for its own requirements. The source node may hold vital data that are crucial for the destination node. This can be accessed by using the value $P_{j,i}^{vital}$. In such instances, the destination node utilizes its own score to offset the negative score of the source node. The destination node resets the updated source score to 0 and deducts the bouncing amount from its own score. Moreover, the SPref is set to MAX, indicating to the source that it does not possess enough score and has been compensated by the destination node. During this process, the destination node also verifies the value of its own score to ensure that it is sufficient for its own future transmissions using ${P(t)}_{i}^{enoughscore}$.

Selfish nodes, which lack scores and social connections with others, find themselves unable to directly send their own data. Despite this limitation, they possess the capability to forward data originating from other nodes, thereby collecting a certain score in return. This mechanism serves as a stimulus for nodes within the network ecosystem. The participation of node i can be accessed by calculating the ${Part}_{i}$.

While the IEEE 802.15.4 standard does not specify a particular packet format for an RRER (Route Reply Error) frame, in conjunction with other protocols, an RRER can also be generated and sent back to the source. Its purpose is to indicate that the source node lacks a sufficient score to proceed with the transmission.

**Figure 2:**
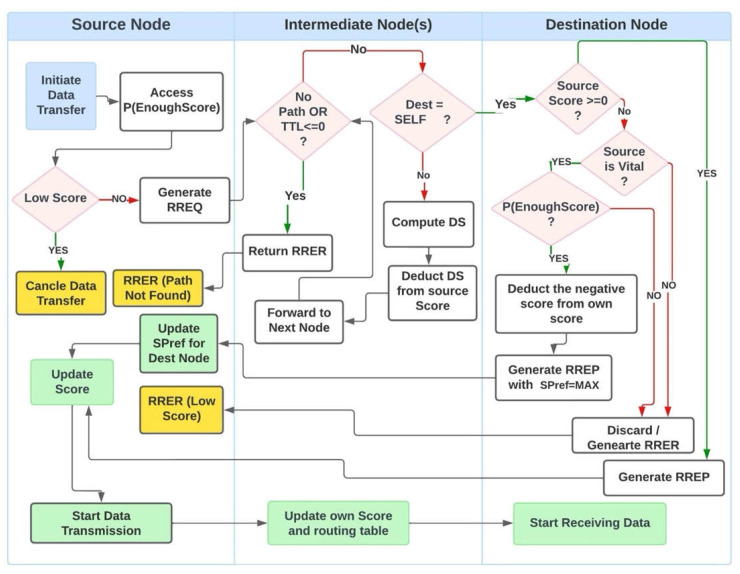
Traffic flow and score management.

The process of score management and transmission is shown in Figure 2.

## 5. Simulation Environment

**Figure 3:**
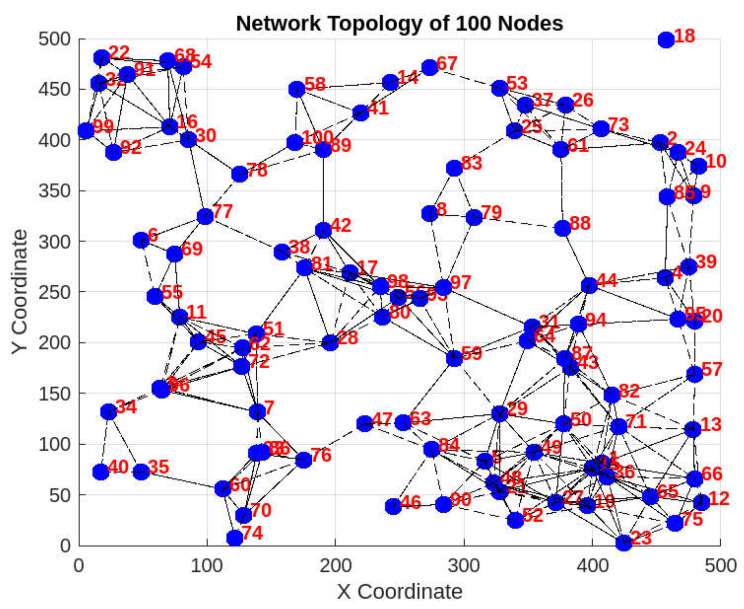
General look of the placement of Nodes.

The work has been simulated in MATLAB 2023b under MacOS Sonoma. Various scenarios have been created with random values for the simulation parameters to assess the resulting values and their impact on network performance. In this section, we discuss one of the scenarios simulated, as shown in Figure 3.

The proposed scheme operates in MATLAB through structured steps, including node initialization, interaction modeling, decision-making, and data transmission. The selection of specific simulation parameters is based on real-world SIoT scenarios to ensure practical applicability. The 500 × 500 m area represents typical smart environments, such as urban sensor networks, smart campuses, or industrial IoT setups, in which nodes are distributed within a confined region. The 100 J energy limit aligns with the constraints of battery-powered IoT devices that ensure a realistic model of energy consumption and depletion patterns. The 512-byte packet size reflects standard IoT communication protocols, in which small-to-medium-sized data packets are used to optimize the transmission efficiency and minimize congestion. Other parameters, such as random node deployment and variable communication ranges, introduce heterogeneity similar to real-world networks, where nodes differ in energy, mobility, and connectivity. These choices enable a comprehensive evaluation of the proposed mechanism under realistic conditions.

The simulation begins by deploying 50 to 300 nodes in the defined area, where each node is initialized with specific attributes, including energy levels (max 100 J), buffer sizes (max 100 bytes), and variable communication ranges. During network operations, nodes periodically identify their CRNs based on their past interactions, proximity, and trust levels. Each node classifies its connected nodes using parameters like transmission history, data quality, energy status, and buffer capacity. Each node assigns an SPref value to its connected nodes based on their historical reliability and computes a DS that reflects their likelihood of being selected as a relay. Nodes with lower DS values are preferred for forwarding to ensure efficient and fair resource utilization. These values dynamically adapt based on the energy consumption, buffer load, and communication performance, allowing the system to remain responsive to network conditions. For reproducibility, the simulation incorporates controlled randomization in node placement and parameter assignment. A fixed random value is applied when necessary to ensure consistency across multiple runs. Multiple scenarios are tested by varying node density (50–300) to assess the robustness of the scheme.

The simulation parameters are listed in Table 4.

Table 5 provides a comprehensive list of a sample of nodes along with their respective energies, buffer sizes, CRNs, the average energy of their CRNs, and earned scores. Each node is also considered with its respective set of CRNs according to the mechanism defined in the previous section. This heterogeneous setup reflects real-world scenarios in which nodes may differ in their capabilities and characteristics, contributing to the complexity of network dynamics.

Table 6 displays the history of transmission sessions, quality of transferred data, preference values, and demanding scores of sample relay nodes (RNode) from their route-requesting source nodes (SNode). It provides sample pairs of nodes within the network.

There are several points to consider when looking at the values in Table 5 and Table 6. Some of these points are listed below.

- A single node can have transmission history with multiple nodes. For example, there is a double entry for RNode 1 with SNodes 41 and 49.
- One node can autonomously adjust its preference levels for other nodes and calculate different DS values for them. Consider the case of SNode 1. A higher preference value is set for RNode 49, i.e., 9.96, which gives it a higher DS than that of RNode 41.
- Consider RNode 10. It has different DS values for SNodes 33 and 42. Another example is the comparison between nodes 10 and 25. Both have almost similar energies (51.21 and 56.56), but they have different DS values for their SNodes due to their different social association, SPref, values.
- Some RNodes are near death, with very little remaining energy, causing significantly higher DS values for their SNodes. Examples include RNodes 20 and 41.
- RNode 10 has a *CRN* node that also impacts the lower DS compared with other nodes. Its *CRN* has a reasonably high energy level. RNode 10 has a very low level of DS for other nodes but has a low value for Data Quality. Consider RNode 78; it also has low Data Quality and low buffer values, directly leading to higher DS values.

These computed values from a particular scenario prove the validity of our work by showing the influence of the considered parameters associated with the nodes’ social, behavioral, and physical characteristics.

## 6. Simulation Results and Discussions

The proposed work is compared with our previous works, reward-based [14], honesty-based [7] and SOS [1]. Our work follows a similar approach to source routing, where each node appends its address to the RREQ and RREP packets. Therefore, we also adopt DSR (Dynamic Source Routing) [38] as the base protocol for performance evaluation. These mechanisms are tuned and assigned with some default parameter values to fit the proposed SIoT network with heterogeneous nodes having varying values, as discussed earlier.

The main performance metrics considered are the packet delivery ratio (PDR), end-to-end delay, throughput, and energy consumption efficiency. Three main scenarios are created with varying numbers of nodes: 500, 100, 200, and 300 nodes. Two types of analyses are performed: time interval-based and varying network size-based. Firstly, the results are recorded for 200 nodes in ten intervals of 100 time pauses, and then the results are recorded for varying numbers of nodes at a time pause of 90. The nodes are randomly deployed in the area.

The network area considered is 1000 × 1000 m^2^. The highest level of energies and buffer sizes are set to 100 J and 100 bytes. The SPre*f* is kept random for all the nodes. The nodes are also programmed to change this value according to their history of data transmission, proximity, and resource levels. Moreover, 25% of the nodes have lower values for SPref. In the case of a reward-based mechanism, the Sel value is kept random to exhibit the random selfishness levels. However, 25% of the nodes are kept selfish by setting Sel at a lower value. In the case of honesty-based and SOS mechanisms, 25% of nodes are kept with selfishness levels. In the case of DSR, 25% of the nodes do not provide relaying services to other nodes. They demonstrate a simple refusal to participate in routine tasks. As the baseline DSR lacks a dedicated mechanism to handle such behaviors, the network performance significantly degrades.

- End-to-End Delays

**Figure 4:**
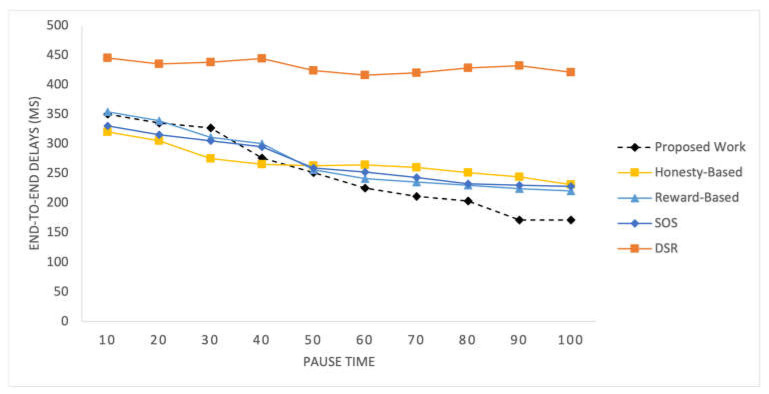
End-to-end delays vs time pauses.

**Figure 5:**
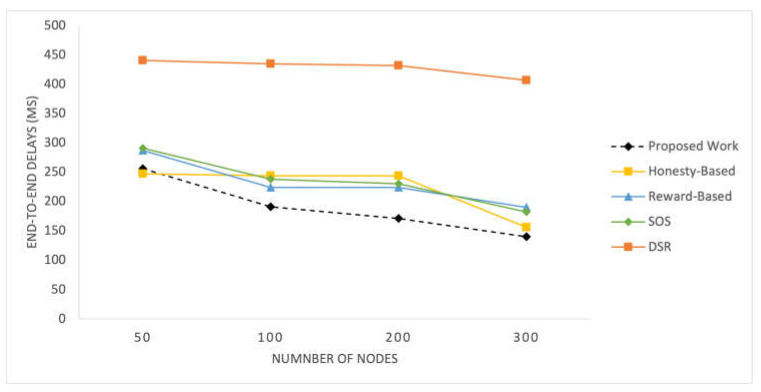
End-to-end delays vs varying number of nodes.

Figure 4 and Figure 5 show the end-to-end delays observed in the SIoT network for different simulation scenarios. The delays are depicted over a range of packet transmission rates and network loads, providing insight into the network’s performance under varying conditions. Figure 4 shows the result for 100 time pauses, while Figure 5 has the results for varying numbers of nodes.

In these figures, it is observed that the end-to-end delays for the proposed work are slightly higher than those for the other mechanisms initially. However, as time progresses, the delays for the proposed mechanism start to decrease and eventually become lower than those for the honesty-based mechanism. In the beginning, the data quality values may not have enough influence on the performance due to the limited traffic records. However, as more traffic flows are observed after a time pause of 40, the delays considerably decrease. The delays peak at a time pause of 90. The DSR exhibits poor performance due to the absence of selfish node management. Conversely, the other mechanisms exhibit a uniform decline in their delays, ultimately resulting in higher values than those of our proposed mechanism.

In the other experiment involving varying numbers of nodes, the proposed method performs similarly to the honesty-based mechanism when there are 50 nodes. However, as the number of nodes increases, the delays are significantly reduced. This improvement can be attributed to the better utilization of social parameters, particularly the data quality indicators used in the mechanism. The reward-based mechanism relies on the density of the nodes in the network; therefore, it performs poorly with a low number of nodes. The honesty-based mechanism initiates the efficient utilization of communities with a higher number of nodes.

B. Throughput

**Figure 6:**
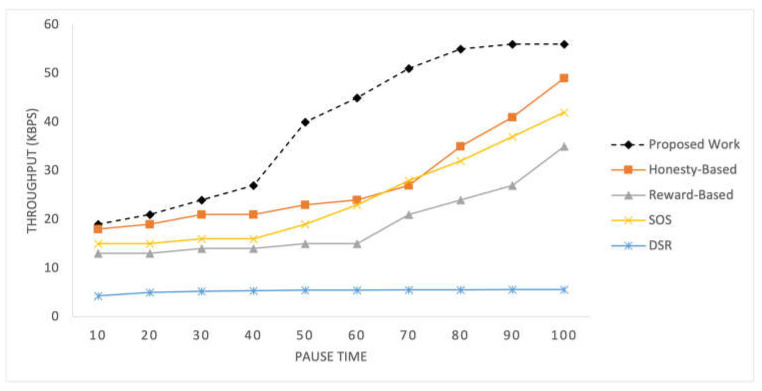
Throughput recorded at 10 intervals.

**Figure 7:**
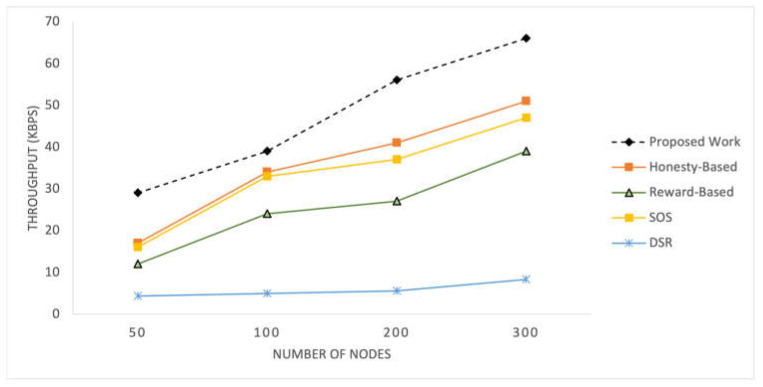
Throughput with varying numbers of nodes in the network.

Figure 6 displays the recorded throughput for the selected mechanisms implemented within the SIoT network for time pauses. Figure 7 shows the throughput results for different numbers of nodes in the network. These graphs present the throughput values achieved by each mechanism under different network conditions, providing a comparative analysis of their performance in terms of the data transmission efficiency.

The throughput of the proposed mechanism is significantly better than that of the compared mechanism. This improvement can be attributed to the adaptive relay selection strategy employed by the proposed mechanism. If a node does not have an efficient score, it refrains from initiating any RREQ, whereas other mechanisms may attempt transfer regardless of their own statuses. Additionally, factors such as mutual social likeness and data quality also contribute to the better results achieved by the proposed mechanism. These factors collectively enhance the efficiency and effectiveness of data transmission in networks. The honesty-based mechanism, similar to the proposed work, utilizes transfer history, represented by interaction frequency, which contributes to achieving better throughput than the reward-based mechanism. By leveraging the transfer history, both mechanisms can make informed decisions regarding relay selection and data transmission, resulting in improved network performance and higher data throughput. SOS exhibits almost similar but slightly lower than the honesty-based mechanism.

With an increase in the number of nodes, all the mechanisms obtain a higher throughput. These mechanisms are highly dependent on the number of nodes because the primary parameters used are related to the number of nodes. With an increased number of nodes, these mechanisms achieve a higher throughput. These mechanisms are highly dependent on the number of nodes because the primary parameters used are associated with the number of nodes. As the network size increases, there are more opportunities for nodes to establish connections and select efficient relay nodes, resulting in improved throughput.

C. Packet Delivery Ratio

**Figure 8:**
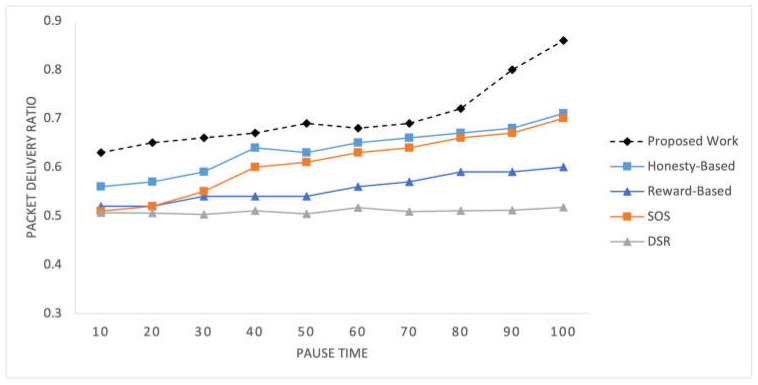
Packet delivery ratio recorded at time intervals.

**Figure 9:**
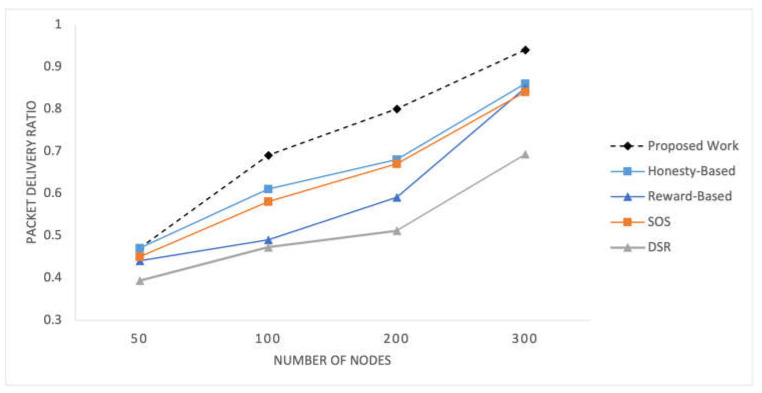
Packet delivery ratio for varying number of nodes in the network.

Figure 8 illustrates the PDR observed within the network for the selected mechanisms at different time pauses. Figure 9 shows the PDR values recorded for networks of varying sizes. These present the percentage of successfully delivered packets to the total number of packets sent, providing a measure of the network’s effectiveness in delivering data packets to their intended destinations.

The social preferences, transmission history, and quality data delivery utilized in the proposed mechanism exert a significant influence the PDR results. As time progresses, nodes can establish better routes, resulting in a consistent increase in the PDR values over different time intervals. Additionally, the number of nodes in the network also influences the PDR, as demonstrated in Figure 9. With a larger number of nodes, more potential routes and relay options are available, leading to variations in the PDR values across different network configurations. The proposed mechanism outperforms this experiment due to the better utilization of social parameters and traffic-associated factors.

D. Energy Efficiency

**Figure 10:**
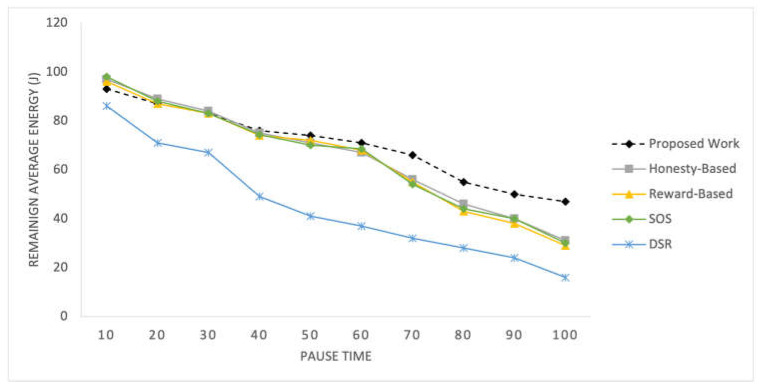
Average remaining energy of nodes recorded at 10 intervals.

**Figure 11:**
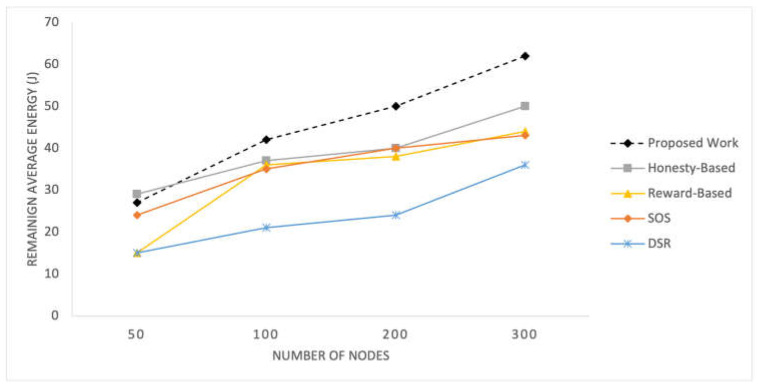
Average remaining energy of nodes for different network sizes.

The results of the competing mechanisms in terms of energy consumption efficiency are presented in Figure 10 and Figure 11 for time pauses and varying numbers of nodes, respectively. These graphs provide a comparative analysis of the energy efficiency achieved by each mechanism, offering insights into their effectiveness in optimizing energy usage in the SIoT network.

Energy consumption is a critical aspect of these networks. In the initial experiment, all mechanisms yield similar results; however, after time pause 60, the proposed mechanism demonstrates slightly better performance in terms of remaining energy levels. These mechanisms rely on the topological and behavioral characteristics of nodes, thereby achieving energy efficiency. When considering varying numbers of nodes, the proposed mechanism consistently exhibits better results. With more nodes, there is a better topological structure and enhanced social associations, leading to balanced energy consumption among all the nodes. The reward-based mechanism is greatly influenced by node density and its importance in the network. Low density results in a higher number of important nodes, leading to increased rewards and more frequent failed RREQs among nodes. Consequently, this mechanism exhibits poor performance in scenarios with 50 nodes.

## 7. Conclusions

For SIoT, a new game based on virtual currency, referred to as scores, is proposed. The primary objective is to address the non-cooperative behavior of self-deciding nodes with social preferences that operate within a network with limited resources. Furthermore, the heterogeneity within such networks is also addressed. In this work, the nodes use their behavioral, social, and topological parameters to design their routing strategies. These parameters include proximity, correlated relays, importance in the network, social preference, and the data transfer history. Additionally, probabilities, such as the probability of vital data, prediction of adequate scores, and quality of data transmission, are employed to improve traffic flows and the management of relaying services. The nodes operate based on the scores during data transmission and relaying. It is important to emphasize that, according to standard network models, end nodes that transmit data from sensors are not designed to function as repeaters. Instead, router nodes (such as access points) are responsible for routing, while relaying is typically managed by switches. Not all nodes in the network can be used to change the route, ensuring that the routing decisions remain efficient and practical. Furthermore, the additional energy costs associated with network exploration and the increased channel load due to queries for studying node energy must be considered.

These scores are utilized to balance the load across all nodes and stimulate selfish nodes, taking into account predefined parameters. The work is simulated, and the results for throughput, latency, energy efficiency, and PDR demonstrate that the proposed mechanism is highly effective and efficient in SIoT environments.

While the proposed routing mechanism effectively incentivizes cooperative behavior and optimizes network performance, it does not currently incorporate a dedicated approach for handling urgent data transmissions. Future research will focus on developing an adaptive prioritization mechanism that allows critical or time-sensitive data to be relayed with higher priority. This can be achieved by introducing an urgency-based score adjustment. Using this approach, the nodes allocate additional resources to forward urgent data while maintaining fairness in the score distribution. Additionally, integrating a dynamic, priority-aware scheduling mechanism will enhance real-time data delivery without compromising network stability. Future work will explore how different social and resource constraints influence urgent data handling within the SIoT environment, ensuring that emergency messages are efficiently propagated while preserving the overall efficiency of the network.

Moreover, the scoring mechanism can be replaced with trust scores. These trust values can be calculated based on social and historical interactions among nodes, allowing for the assessment of node credibility within the network. Additionally, different levels of selfishness can be defined, and appropriate management techniques can be proposed to address them.

## Figures and Tables

**Table 1:** Parameters Affecting the Demanding Score.

Parameter	Impact
Energy	The energy of a node has an inverse relationship with the value of its DS. A node having less amount of energy will have a higher value of DS for many source nodes and vice versa.
Buffer Size	Just like energy, the buffer size of a node has also an inverse relationship with the value of DS.
Data Sharing History	Nodes having frequent data sharing or larger data transfer leads to a higher level of DS. This is made so that nodes can share data with any other node in the network, and strong node affiliations are discouraged.
More CRNs	The higher number of CRNs or their energies leads to a lower value of DS. If a node has co-related relays, it can preserve its energy by not taking any request.
Selfishness/ Preference	Nodes can adaptively set their own preference levels. This value can highly influence the computation of DS. This value can be used to exhibit the likeness or dislikeness of a node.
Quality of data transmission	Low-quality data transmissions lead to low DS. This is because there are chances of failed transmissions, and may need to retransmit the data. Nodes having poor connections are discouraged from transferring data to each other.

**Table 2:** Format of modified RREQ Packet.

Field	Size
Frame Control (FC)	1 Byte
Frame Length	1 Byte
Source Address	8 Bytes (may vary)
Destination Address	8 Bytes (may vary)
Hop Count	1 Byte
Route Request ID	4 Bytes
Frame Check Sequence	2 Bytes
Source Score	4 Bytes

**Table 3:** Format of modified RREP Packet.

Field	Size
Fields similar to RREQ packet	25 Bytes
Lifetime	2 Bytes
*SPref*	2 Bytes
Updated Source Score	4 Bytes

**Table 4:** List of parameters used in simulation.

Parameter	Value
Simulation Tool	MATLAB 2023b
Operating System	MacOS Sonoma
Simulation Area	500 × 500 square meters
Number of Nodes	50–300
Node Deployment	Random
Node Heterogeneity	Energy, Buffer Size, Communication Range
Maximum Node Energy	100 Joules
Maximum Buffer Size	100 Bytes
Communication Range	Variable among nodes
Routing Mechanism	Socially Inspired Node Stimulation Scheme
Mobility Model	Random Waypoint Model
Transmission Power	Adaptive
Traffic Type	Constant Bit Rate (CBR)
Packet Size	512 Bytes
Simulation Time	100 s
Performance Metrics	Energy Efficiency, Throughput, PDR, End-to-End Delay

**Table 5:** List of nodes with values.

N-ID	Energy	B-Size	*CRNs*	*Avg*	Score
1	84.39	3.1	1.63	84.72	74.79089
2	49.01	64.48	2	49.01	26.95842
10	51.21	57.54	10.89	66.84	176.6978
19	17.58	5.31	18	17.58	211.8241
20	4.3	6.39	20	4.3	4212.113
27	48.34	52.63	27	48.34	19.65204
33	34.79	36	33	34.79	241.1593
41	0.39	71.8	41	0.39	56,427.56
46	67.3	49.18	46	67.3	133.6194
49	15.93	51.7	49	15.93	269.5232
59	51.38	35.28	59	51.38	169.2944
68	3.09	58.68	42.68	40.52	4371.384
78	65.83	7.7	78	65.83	132.4351
82	8.38	84.97	82	8.38	3991.539
93	35.52	68.98	93	35.52	197.3984
99	9.03	83.33	99	9.03	3841.583

**Table 6:** Calculated values for demanding scores.

RNode	SNode	History	Quality	SPref	DS
1	41	1	0.72	3.25	0.010162
1	49	10	0.72	9.96	0.311417
2	4	43	0.81	4.56	0.156735
10	33	18	0.06	5.02	0.380622
10	42	32	0.06	9.05	1.219877
19	66	10	0.79	5.69	4.383997
19	95	42	0.79	4.27	13.817682
20	5	44	0.7	6.47	342.66789
20	65	22	0.7	7.23	191.45972
27	80	4	0.56	9.42	0.054337
33	41	11	0.28	9.20	0.832671
33	84	20	0.28	3.90	0.641782
41	1	14	0.48	6.15	1603.9471
41	33	39	0.48	9.05	6575.0657
46	11	3	0.32	4.62	0.019751
49	1	34	0.68	8.73	3.310108
59	14	19	0.97	8.25	0.173959
68	28	28	0.69	7.33	2.011814
78	3	47	0.06	5.12	12.70449
78	58	21	0.06	9.46	10.48818
82	30	50	0.93	2.35	2.106026
93	80	40	0.2	9.21	2.082972
99	96	5	0.25	2.05	0.59894

## Data Availability

Data are contained within the article.
